# Structures and Functions of Pestivirus Glycoproteins: Not Simply Surface Matters

**DOI:** 10.3390/v7072783

**Published:** 2015-06-29

**Authors:** Fun-In Wang, Ming-Chung Deng, Yu-Liang Huang, Chia-Yi Chang

**Affiliations:** 1 School of Veterinary Medicine, National Taiwan University, No. 1, Section 4, Roosevelt Road, Taipei 10617, Taiwan; E-Mail: fiwangvm@ntu.edu.tw; 2 Animal Health Research Institute, Council of Agriculture, 376 Chung-Cheng Road, Tansui, New Taipei City 25158, Taiwan; E-Mails: mcdeng@mail.nvri.gov.tw (M.-C.D); ylhuang@mail.nvri.gov.tw (Y.-L.H)

**Keywords:** pestivirus, glycoprotein, E^rns^, E1, E2, structure, function

## Abstract

Pestiviruses, which include economically important animal pathogens such as bovine viral diarrhea virus and classical swine fever virus, possess three envelope glycoproteins, namely E^rns^, E1, and E2. This article discusses the structures and functions of these glycoproteins and their effects on viral pathogenicity in cells in culture and in animal hosts. E2 is the most important structural protein as it interacts with cell surface receptors that determine cell tropism and induces neutralizing antibody and cytotoxic T-lymphocyte responses. All three glycoproteins are involved in virus attachment and entry into target cells. E1-E2 heterodimers are essential for viral entry and infectivity. E^rns^ is unique because it possesses intrinsic ribonuclease (RNase) activity that can inhibit the production of type I interferons and assist in the development of persistent infections. These glycoproteins are localized to the virion surface; however, variations in amino acids and antigenic structures, disulfide bond formation, glycosylation, and RNase activity can ultimately affect the virulence of pestiviruses in animals. Along with mutations that are driven by selection pressure, antigenic differences in glycoproteins influence the efficacy of vaccines and determine the appropriateness of the vaccines that are currently being used in the field.

## 1. Introduction

The genus *Pestivirus* belongs to the family *Flaviviridae*, which also includes the genera *Hepacivirus* and *Flavivirus* [1]. Pestiviruses include economically important animal pathogens, such as bovine viral diarrhea virus (BVDV), classical swine fever virus (CSFV), and border disease virus [2]. BVDV and CSFV are closely related at the structural, antigenic, and genetic levels. Pestiviruses are enveloped RNA viruses containing single-stranded, positive-sense RNA genomes of approximately 12.3–12.5 kb [3] that consist of a single open reading frame encoding a single polyprotein of approximately 4000 amino acids flanked by a 5′ untranslated region (UTR) and a non-polyadenylated 3′UTR [4]. The translated polyprotein is processed co- and post-translationally by viral as well as host cellular proteases into mature viral proteins, including four structural and eight nonstructural proteins in the order NH_2_-N^pro^-C-E^rns^-E1-E2-p7-NS2-NS3-NS4A-NS5B-COOH [4].

The structural components of the virion include nucleocapsid protein C and three envelope glycoproteins: E^rns^, E1, and E2 [5]. Glycoprotein processing is initiated by nascent cleavage between the capsid protein and the precursor E^rns^E1E2, which is followed by cleavage at the *C*-terminal end of E2. E^rns^E1E2 is cleaved to form E^rns^E1 and E2, and then E^rns^E1 is processed into E^rns^ and E1 [6]. The cleavage between E^rns^ and E1 and that between E1 and E2 are both catalyzed by a host signal peptidase within the lumen of the endoplasmic reticulum (ER) [7]. E1 and E2 contain transmembrane domains (TMD), whereas E^rns^ lacks a TMD and instead anchors to the membrane in a unique manner, and is also secreted from infected cells [6]. All three glycoproteins form disulfide-linked complexes: the E^rns^ homodimer, E1-E2 heterodimer, and E2 homodimer [5]. Glycoprotein E2 is the major envelope protein exposed on the outer surface of the virions and induces neutralizing antibody responses during CSFV and BVDV infections [8]. E^rns^ is a second glycoprotein that mediates neutralization [9,10] and has the unique feature of possessing intrinsic ribonuclease (RNase) activity [11,12]. Of the three glycoproteins, the functions of E1 are the least well understood.

To further illuminate the roles of these pestivirus glycoproteins in viral replication, viral interactions with hosts and cells, immune response elicitation, and viral pathogenicity, this article summarizes the structures and functions of E^rns^, E1, and E2 and discusses how they affect pestivirus virulence in infected cells and animals.

## 2. Structures of the Pestivirus Glycoproteins

### 2.1. Crystal Structures of the Glycoproteins

E^rns^ has a mass of 44–48 kDa [5], and its *C*-terminus functions as (1) a membrane anchor; (2) a retention/secretion signal; (3) a binding site for cell surface glycosaminoglycans (GAGs); and (4) a signal peptidase cleavage site. In contrast, the *C*-terminus of E^rns^ lacks a transmembrane helix [6]; E^rns^ instead anchors to the membrane via its *C*-terminus, which folds into an amphipathic helix [13,14]. A recent study using circular dichroism and nuclear magnetic resonance spectroscopy revealed that the membrane anchor of E^rns^ is comprised of a domain that spans 61 residues, K^167^-A^227^, which forms a continuous amphipathic α-helix. Both the hydrophilic and hydrophobic faces of the helix are maintained throughout its entire length, suggesting that it could bind in a flat manner onto the membrane surface. A central stretch of 15 residues that is fully shielded from the aqueous layer has also been identified within the helix; this region is followed by a putative hairpin structure at the *C*-terminus. These findings explain the possible mechanisms behind E^rns^’s contact with the membrane, its processing, and its secretion [15]. Because of the unusual membrane anchor at its *C*-terminus, a significant portion of E^rns^ is secreted [6]. The *C*-terminus is also necessary for the retention/secretion of E^rns^ [13,14,16], with residues L^183^, I^190^, and L^208^ being important for its intracellular localization [16]. Additionally, a binding site for cell surface GAGs is located within the *C*-terminal region of E^rns^, in which a cluster of basic residues, ^213^KKLENKSK^220^, and a mutation (S^209^R) are crucial for heparan sulfate (HS) binding [17,18]. The *C*-terminus of E^rns^ is formed upon cleavage of the E^rns^E1 precursor protein by a cellular signal peptidase [7].

The crystal structure of the catalytic domain of E^rns^, which has RNase activity, has been recently elucidated. The domain contains one folding and two binding sites for substrate recognition, corresponding to T2 RNase from plants and fungi [19]. The active site of BVDV E^rns^ comprises H^32^, H^76^, E^77^, K^80^, and H^81^, and single-site mutations can render the enzyme catalytically inactive [20,21]. Within this site, H^81^ of E^rns^ acts as a catalytic base and is stabilized by H^76^, E^77^, and K^80^; H^32^ acts as a hydrogen donor to release the cleavage products [19].

Among the three pestivirus glycoproteins, E1 is the least studied. E1 has a mass of 33 kDa [5] and is classified as a type I transmembrane protein [8]. The *N*-terminus of E1 is an ectodomain, and the *C*-terminal region harbors a hydrophobic anchor that attaches E1 to the envelope of the virus [5]. The E1 membrane anchor contains two amphipathic perimembrane helices and one transmembrane helix [22]. No crystal structure information on the E1 protein is available to date.

**Figure 1:**
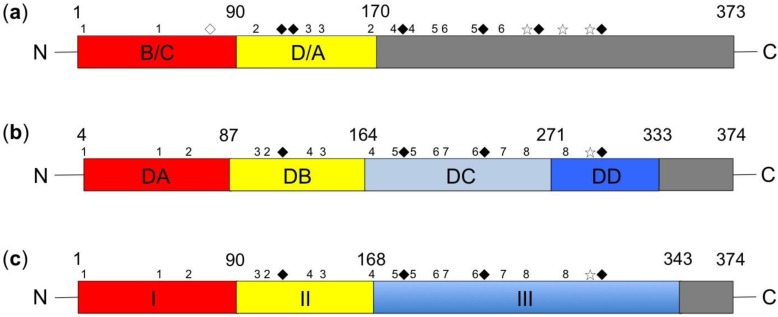
CSFV and BVDV E2 glycoproteins possess remarkably similar topologies when mapped using different approaches. (**a**) The domains of CSFV E2 modified from Chang *et al.* [25,26]. Domains B/C, comprising residues 1–90, are in red, and domains D/A, comprising residues 91–170, are in yellow; (**b**) The domains of BVDV E2 modified from El Omari *et al.* [29]. Domain DA, comprising residues 4–87, is in red, and domain DB, comprising residues 88–164, is in yellow. Domain DC, comprising residues 165–271, is in light blue, and domain DD, comprising residues 272–333, is in dark blue; (**c**) The domains of BVDV E2 modified from Li *et al.* [30]. Domain I, comprising residues 1–90, is in red, and domain II, comprising residues 91–168, is in yellow. Domain III, comprising residues 169–343, is in medium blue. The residue numbers are shown above the schematics. The cysteines involved in intramolecular disulfide bridges are marked by numbers sequentially according to disulfide bonds, whereas the cysteines involved in intermolecular disulfide bridges are marked by white asterisks. N-linked glycosylation (NLG) sites are denoted by black diamonds, and an O-linked glycosylation (OLG) site is denoted by a white diamond.

E2 has a mass of 55 kDa [5] and is classified as a type I transmembrane protein. It has an *N*-terminal ectodomain and a hydrophobic domain in its *C*-terminus that anchors into the viral envelope [5]. The E2 anchor contains a short helical hairpin that is stabilized in the membrane by an arginine residue, similar to what has been found in flaviviruses [22]. For CSFV E2, four domains have been identified in the order of B/C/D/A (Figure 1a) [23,24]. All four domains are located in the *N*-terminal half of E2 and constitute two independent structural units, a unit comprised of domains B/C (residues 1–90) and a unit comprised of domains D/A (residues 91–170) [25,26,27,28]. Domains B/C and domains D/A each form an Ig-like fold with a panhandle structure that is linked via the intervening region [27,28]. The *C*-terminal domain forms a single β-sheet.

Recently, the crystal structures of the E2 protein of BVDV have been solved [29,30,31]. One study divided E2 into four domains (Figure 1b) [29]. Domains DA and DB (residues 4–87 and 88–164, respectively) are the most distal from the viral membrane and are likely to be the most exposed on the virus surface. Both domains possess Ig-like folds. Domain DC (residues 165–271) is a highly extended disulfide-rich structure that is composed of loops and antiparallel β strands. Domain DD (residues 272–333) is the most conserved domain among pestiviruses.

Another study divided E2 into three domains (Figure 1c) [30]. Domain I (residues 1–90) is an Ig-like domain. Domain II (residues 91–168) is a seven-stranded Ig-like domain with an overall shape and size similar to that of domain I. A sequence of 12 residues between the last two β strands in domain II forms a highly exposed β hairpin that protrudes into the solvent. Domain III (residues 169–343) consists of a series of three small β-sheet modules (IIIa-c), which together form an elongated domain.

The E2 proteins of CSFV and BVDV have been mapped as having similar topologies. Domains DA and DB of BVDV correspond to CSFV domains B/C and D/A, respectively [29]. In another study, domain I was found to correspond to CSFV domains B/C, and domain II mapped to CSFV domains D/A. Domain III does not harbor any antibody epitopes, suggesting that it is not exposed on the viral surface [30]. Collectively, the above studies reveal that the structure of BVDV E2 is similar to that of CSFV E2 (Figure 1).

### 2.2. Intramolecular Disulfide Linkage and Dimerization of Glycoproteins

The glycoproteins form disulfide-linked complexes, including an E^rns^ homodimer of 97 kDa, an E1-E2 heterodimer of 75 kDa, and an E2 homodimer of 100 kDa [5].

The E^rns^ protein contains nine cysteines, eight of which form four separate intramolecular disulfide bonds [32], as confirmed by recently reported crystal structures [19]. The four intramolecular disulfide bonds are strictly conserved across all members of the genus *Pestivirus*, indicating the importance of these disulfide bonds in protein folding and/or function. The ninth cysteine residue, C^171^, although not directly involved in a disulfide linkage, is essential for the homodimerization of E^rns^ and can influence virulence [32,33]. Mutation of C^171^ results in a loss of the dimeric state of E^rns^ and reduces its binding affinity for HS, suggesting that the E^rns^ homodimer is crucial for HS binding [34]. Because C^171^ is not conserved among pestiviruses, it has been suggested that the E^rns^ homodimerization linkage via this cysteine residue might not be essential for pestivirus viability.

The E1 protein contains six cysteines. In the formation of the E1-E2 heterodimer, substitutions of C^24^ and C^94^ in CSFV E1 affect the formation of E1-E2 heterodimers and alter virulence [35]. The charged residues K^174^ and R^177^ in the TMD of E1 and R^355^ in the TMD of E2 play key roles in E1-E2 heterodimerization [36]. A recently constructed heterotetrameric model of the E1-E2 assembly suggested that residue C^171^ in E1 forms a disulfide bond with residue C^295^ in E2, thereby stabilizing the E1-E2 interaction that is required for virus infectivity. The model further confirmed that these charged residues are crucial for E1-E2 heterodimerization [36] because they promote disulfide bonding between E1 C^171^ and E2 C^295^ [22].

CSFV E2 contains 15 cysteines. An intramolecular disulfide bond forms the structural unit of domains B/C, anchored via a disulfide bond between residues C^4^ and C^48^. The structural unit of domains D/A is formed by two disulfide bonds, one between residues C^103^ and C^167^ and the other between C^129^ and C^139^ [27]. In the formation of the CSFV E2 homodimer, the last three cysteines at residues C^256^, C^277^, and C^294^ in the *C*-terminus function in mediating homodimerization [26] (Figure 1a). In BVDV E2, all 17 cysteines are involved in disulfide bridges, establishing one inter- and eight intramolecular bonds. The 17th cysteine at residue C^295^ forms an intermolecular bond with the dimer partner molecule [29,30] (Figure 1b,c). Although the cysteine residues of BVDV are highly conserved, the E2 protein of BVDV, unlike that of CSFV, contains two additional cysteine residues, C^59^ and C^106^. Although the exact functions of these additional cysteines remain unclear, they may be involved in alternative disulfide bonds, thus affecting the structure of the E2 protein of BVDV in a different manner than that of CSFV.

### 2.3. N- and O-Linked Glycosylation of Glycoproteins

Glycosylation is one of the most common types of protein modification, whereby N-linked oligosaccharides are added to specific asparagine residues within the context of the consensus sequence Asn-X-Ser/Thr [37]. The CSFV E^rns^ protein has a high degree of N-linked glycosylation (NLG) and contains seven sites, at residues 2, 7, 11, 65, 95, 143, and 158, contributing to nearly half of the molecular mass of the protein [38,39]. The CSFV E1 protein has three putative NLG sites, at residues 6, 19, and 100 [40]; CSFV E2 contains one putative O-linked glycosylation (OLG) site at residue 75 and six NLG sites at residues 116, 121, 185, 229, 260, and 297 [41] (Figure 1a). BVDV E2 has four NLG sites at residues 117, 186, 230, and 298, one of which is in domain DB, two in domain DC, and one in domain DD [29] (Figure 1b); alternatively, one is located in domain II and three in domain III [30] (Figure 1c).

*N*-glycosylation may play a role in the transport of E^rns^ through the secretory pathway [38] and may also influence glycoprotein dimer formation, synthesis, and processing [42]. N-glycan moieties on E^rns^ are essential for its ability to bind to double-stranded RNA and to block the induction of interferon (IFN) [43]. Furthermore, the glycosylation statuses of E^rns^, E1, and E2 affect virulence, as the abolishment of specific NLG sites leads to virus attenuation [39,40,41]. It has been suggested that glycosylation patterns play roles in viral attachment, entry, and/or exit from infected cells [41].

The glycosylation patterns of E^rns^ and E2 affect the induction of immune responses. Neutralizing epitopes of the E^rns^ and E2 proteins are dependent on the presence of glycosylation. Indeed, preventing proper post-translational glycosylation of E^rns^ and E2 has been shown to lead to the synthesis of non-immunogenic proteins and to failure to induce protection against CSFV [44].

### 2.4. Antigenic Structure and Epitopes of Glycoproteins

Although the biochemical and functional properties of the E^rns^ protein have been well characterized, relatively little is known about its antigenic structure, with only linear epitopes being identified thus far. Peptides comprising *C*-terminal residues 191–227 of E^rns^ are immunogenic when applied as ELISA antigens [45]. By deletion analysis, three overlapping regions, at amino acid positions N^65^-S^145^, W^84^-S^160^, and E^109^-K^220^, have been identified as antigenic regions that can be recognized by pig anti-CSFV sera [46]. Five linear epitopes of E^rns^—^31^GIWPEKIC^38^, ^65^NYTCCKLQ^72^, ^127^QARNRPTT^134^, ^145^SFAGTVIE^152^, and ^161^VEDILY^166^—have been mapped and found to contain the conserved residues W^33^, L^71^, Q^127^, N^130^, S^145^, and G^148^, which may be critical for antibody binding [47]. Additional linear epitopes, including ^114^CRYDKNTDVNV^124^ and ^116^YDKNTDVNV^124^, have been identified [48] as containing the binding motif ^117^DKN^119^ [49]. The domain comprises three linear motifs, ^64^TNYTCCKLQ^72^, ^73^RHEWNKHGW^81^, and ^88^DPWIQLMNR^96^, which are also defined, and two residues, T^102^ and D^107^, are crucial for interactions with antibodies [50].

Conversely, the antigenic structure and epitopes of the E1 glycoprotein are still not resolved and remain to be investigated.

For CSFV, the antigenic structure of E2 and its epitopes have been extensively studied. Domains B/C, in which non-conserved epitopes are located, are responsible for antigen specificity among various CSFV strains, whereas domains D/A are relatively conserved [25,51]. Several important conformational epitopes have been defined within E2 [25,26,27,52,53,54,55]. To achieve the correct folding of its four antigenic domains, all of the conformational epitopes of E2 depend on the pairing of six different cysteine residues located in the *N*-terminal half of the protein [25,26,27] (Figure 1a). In domains B/C, the neutralizing antigenic motifs ^64^RYLASLHKKALPT^76^ and ^82^LLFD^85^ are essential for maintaining the structural integrity of conformational epitopes [25,53], with residues E^24^ and D^40^ being responsible for the antigenic specificities of field strains and residues D^16^ and K^72^ being responsible for specificity of the LPC vaccine strain [54]. Neutralizing epitopes are also present in domains D/A, and residue R^156^ is responsible for the antigenic specificities of different CSFV genotypes [26]. Regarding the *C*-terminal half of E2, five proximal cysteines at positions 180, 188, 204, 207, and 241 are critical for the structural integrity of the *C*-terminal conformational epitopes [26]. A neutralizing conformational epitope with the antigenic determinant residue E^213^ is also present in the *C*-terminal region [26].

Several important linear epitopes [49,56,57,58,59,60] are present within E2; however, no linear epitope has been identified within domains B/C. A linear epitope is present at motif ^83^LFDGTNP^89^, which borders domains B/C and A [56,57]. In domain A, a highly conserved neutralizing linear epitope, ^140^TAVSPTTLR^148^ [58], which was identified by mAb WH303, has been used to develop epitope-based vaccines [61,62,63,64,65,66] and in serodiagnosis [67]. In the *C*-terminal end of E2, the linear epitope ^306^YYEP^309^ is highly conserved among pestiviruses [60].

Despite several studies that have employed epitope mapping using mAbs, no antigenic structural model is available for BVDV E2 thus far. For genotype 1, amino acid positions that are essential for neutralization have been mapped to the *N*-terminal half of E2 [68]. For genotype 2, three separate neutralizing antigenic domains on E2 have been defined via binding competition assays, but these domains have not yet been mapped [69]. It is presumed that the E2 proteins of both BVDV genotypes exhibit comparable antigenic structures with type-specific epitopes [70]. The immunodominant region of BVDV E2 spans residues 71–74 and includes a key residue at position 72 [68,71]. An antigenic motif in CSFV E2 has also been mapped within this region [53], and residue 72 is also an antigenic determinant [54]. Additionally, a common epitope among pestiviruses has been mapped to domain B in CSFV E2 [55].

## 3. Functions of Glycoproteins during the Pestivirus Life Cycle

During the life cycle of a virus, there are a multitude of functions that glycoproteins must fulfill to enable the virus to successfully infect cells or animals and to subsequently replicate and successfully exit the infected cells (Table 1). These functions can be classified into the following three mutually related components: interacting with cells to infect and replicate, interacting with hosts to maintain itself within the animal population, and interacting with fellow viral proteins to form viable virions.

**Table 1:** Functions of the pestivirus glycoproteins during the viral life cycle.

Category	Functions	References
Interactions with cells	Attachment: E^rns^ and E2	[72]
	Entry: E1 and E2	[36,72,73]
	Cultured cell tropism and host specificity: E2	[74,75,76]
	Interactions with cellular receptors: E^rns^ (heparan sulfate, laminin receptor) and E2 (CD46, heparan sulfate)	[77,78,79,80,81,82,83,84]
	Interactions with cellular proteins: E2	[85,86,87,88]
	Fusion: E2 (CSFV) and E1 (BVDV)	[22,29,30,89,90,91,92,93,94]
	Endocytosis: E^rns^	[91,92,95]
	Autophagy: E^rns^ and E2	[96,97,98]
Interactions with other viral proteins	Dimerization: E1-E2 heterodimer (major), E^rns^ homodimer, and E2 homodimer	[5]
	Virion packaging and assembly: E2 homodimer early and then E1-E2 heterodimer later	[99]
Functions in pathogenesis	Interactions with receptors to determine cell tropism and pathogenicity	[74,75]
	Eliciting host humoral immunity: E2 induces the major neutralization antibody, and E^rns^ induces the second neutralization antibody	[8,9,10]
	Eliciting host cellular immunity: E2 is the target of CTL, and E^rns^ and E1 also have roles	[100,101,102]
	Evasion from immunity: RNase activity of E^rns^ induces apoptosis and inhibits IFN synthesis; E^rns^ and E2 are responsible for positive selection	[43,103,104,105,106,107,108,109,110,111]
	Virulence: E^rns^, E1 and E2	[21,33,35,39,40,41,112,113,114,115,116,117,118,119,120,121]

### 3.1. Interactions with Cells

Cellular attachment and entry is the first step of viral infection of host cells. E^rns^ plays a role in virus attachment [72], whereas E2 is involved in both virus attachment to and entry into target cells [72], thereby determining the cell tropism of pestiviruses [74,75]. The sequences and structures of E2 proteins are presumed to be involved in pestivirus host specificity at the level of cell entry [76]. E1 and E2 form an E1-E2 heterodimer, which is located in the viral envelope and mediates viral attachment and entry [36,73], whereas E^rns^ appears to be dispensable to the process of cell entry. Moreover, three different positively charged residues, two in the E1 TMD and one in the E2 TMD, are essential for cell entry [36], suggesting that interactions within the E1/E2 TMD complex are essential for pestivirus entry into cells.

E^rns^ and E2 glycoproteins interact with different cell surface receptors [72]. Cell surface GAGs, such as HS, can serve as receptors for E^rns^ [77,78]. A recent study has demonstrated that the laminin receptor (LamR) is a cellular attachment receptor for CSFV E^rns^ [79]. LamR operates as an alternative pathway to the HS pathway [79]. These two molecules are also associated with infection by dengue virus, which is another member of the *Flaviviridae* family, suggesting that CSFV and dengue virus may use similar mechanisms during viral entry [79]. The cell surface receptor of the BVDV E2 glycoprotein is bovine CD46 [80], and inhibition of BVDV infection by CSFV E2 suggests that CSFV E2 and BVDV E2 share an identical receptor [72]. Indeed, both porcine CD46 and HS were recently shown to be the primary components that drive CSFV attachment and entry [81]. The function of CD46 as a cellular receptor for BVDV is modulated by complement control protein 1 (CCP1), which subsequently promotes entry of the virus [82], and genetic and splice variants of CCP1 determine cell permissivity [83]. The BVDV receptor-binding sites of CD46 have been mapped to two peptides, ^66^EQIV^69^ and ^82^GQVLAL^87^, which are located on antiparallel β-sheets in CCP1. These two peptides constitute a crucial region of a binding platform that interacts with BVDV [82]. The potential host cell binding sites of pestivirus are the regions ^101^LAEGPPVKECAVTCRYDKDADINVVTQARN^130^ of E^rns^ and ^141^AVSPTTLRTEVVKTFRRDKPFPHRMDCVTT^170^ of E2 [84].

Cellular β-actin interacts with E2 and functions in both entry and the endocytic pathway [85]; it is also involved in the early replication of CSFV [86]. The domain of β-actin that interacts with E2 has been mapped to amino acids 95–188, and its counterparts on E2 have been mapped to two regions that include amino acids 182–261 and 262–341 [86]. Recently, the cellular membrane protein annexin 2 (Anx2) has been identified as a cellular binding protein for CSFV E2 and shown to impact CSFV attachment and entry, RNA replication, and virion production [87]. A study in swine cells using a yeast two-hybrid system identified several proteins that might serve as potential host binding partners, interacting with a non-linear portion of CSFV E2 [88]. Because many of the identified host proteins are also involved in interactions with other viruses, it has been suggested that these proteins might play a role in pestivirus replication or pathogenesis [88].

Cellular entry of enveloped animal viruses requires fusion between the viral and cellular membranes. E2 is characterized as a class II fusion protein that harbors a fusion peptide, ^129^CPIGWTGVIEC^139^, containing a consensus sequence comprised of aromatic and hydrophobic residues between two cysteine residues [89]. As has also been recently confirmed, the fusion peptide is involved in membrane fusion activity and has a critical role in virus replication [90]. BVDV E1 contains a fusion motif, whereas E2 acts as a structural scaffold for E1 [29,30]. Pestivirus entry is dependent on clathrin-mediated endocytosis [91,92], and acidification initiates fusion [93]. During endocytosis, low pH triggers conformational changes that result in insertion of the fusion peptide into the target membrane. In fusion proteins, histidine is assumed to play a role in pH-induced conformational changes [94], and a histidine at residue 70 of BVDV E2, which is conserved among pestiviruses, is exposed on the surface of the domain at the membrane-distal end of the molecule; it may trigger order–disorder transition of this domain at low pH [29]. A recent study has proposed that two different juxtamembrane residues, H^335^ and H^336^, might also participate in a pH-sensing mechanism in BVDV [22].

Recent studies have demonstrated that pestivirus infection significantly induces cell autophagy [96,97], which promotes viral replication and maturation *in vitro* [97]. It has been further identified that the E^rns^ and E2 proteins serve as important regulators in autophagy pathways; conversely, E1 is not involved in this process [98].

### 3.2. Interactions with Other Proteins within Virions

Pestivirus glycoproteins interact with each other by forming disulfide-linked complexes, namely, the E^rns^ homodimer, E1-E2 heterodimer, and E2 homodimer [5]. E1-E2 heterodimers are thought to be a major complex in mature virions [8]. During virus assembly, E2 homodimers are formed early, and E1-E2 heterodimers are formed later, after the release of E1 from the ER chaperone calnexin [99]. The dimerization of pestivirus glycoproteins indicates that intact disulfide bonds are critical for acquiring a stable conformation of E2 monomers [122]. Forcing E2 to adopt a reduced conformation during the process of virus maturation results in protein misfolding and proteasome degradation. In contrast, dimerization of E2 results in a conformation that is resistant to reducing agents and degradation. Furthermore, E1-E2 heterodimers are essential for viral entry and infectivity [36].

### 3.3. Interactions with the Host

Pestivirus glycoproteins can elicit both humoral and cellular immune responses in a host. As discussed above, E2 functions as a major antigen that can elicit neutralizing antibody production that confers protection to the host [8], whereas E^rns^ functions as a secondary antigen during infection [9,10]. Thus, serological diagnoses of CSFV-infected animals are primarily based on the detection of E2- or E^rns^-specific antibodies [123,124,125]. E2 has also been identified as a target for T-cell activation, which is important for targeting cytotoxic T-lymphocyte (CTL) responses [100,101,102]. As they contain several defined T-cell epitopes, both E^rns^ and E1 proteins have been identified as targets for the cellular immune response [100].

In the family *Flaviviridae*, the structural E^rns^ protein is unique to pestiviruses [2] in that it harbors an RNase active domain of the T2 RNase superfamily [12]. Monoclonal antibodies (mAbs) that inhibit the RNase activity of E^rns^ tend to neutralize virus infectivity, which suggests that the RNase activity of E^rns^ plays a role in the CSFV life cycle [126]. This RNase activity of E^rns^ can induce apoptosis in lymphocytes [103] and can block the synthesis of type I IFN, which is induced by viral single-stranded and double-stranded RNA [104,105]. As the blockage of IFN induction occurs during the initial step of Toll-like receptor 3 signaling [43], pestivirus E^rns^ plays a central role in evading the host’s IFN response and favors the establishment and maintenance of persistent infection [106]. Furthermore, a previous study has demonstrated that the RNase activity of E^rns^ can prevent the activation of plasmacytoid dendritic cells (pDCs) by CSFV-infected cells [107]. As pDCs are the most potent source of type I IFN during the early phases of viral infection, this important finding identifies a novel pathway by which viruses can escape the IFN system.

Recently, the localization of E^rns^ and the mechanism leading to evasion of host innate immunity have been further examined. E^rns^ is taken up by a cell within minutes via clathrin-mediated endocytosis, and this uptake is largely dependent on its *C*-terminus, which binds to cell surface GAGs. The inhibitory activity of E^rns^ remains for several days, indicating its potent and prolonged effect as a viral IFN antagonist [95]. 

It is likely that E2 serves as the major pestivirus protein that interacts with cell receptors, whereas E^rns^ serves as an accessory protein that interacts with other cell surface molecules, which in a sense resemble co-stimulatory molecules, to deliver appropriate signaling for viral entry or endocytosis. Inappropriate signaling would likely alter viral replication and govern whether a productive or a persistent infection will ensue.

## 4. The Roles of Viral Glycoproteins in Pathogenicity in Animals or Cells

### 4.1. Genetic Basis of Pestivirus Virulence

The molecular determinants of pestivirus virulence have been defined by reverse genetic technology [112]. It appears that seven proteins of pestiviruses, including its three glycoproteins, play roles in virulence [113]. As will be reviewed below, the majority of events that occur on the surface of viable virions, such as the RNase activity of E^rns^, variations in amino acids and antigenic structures, and altered patterns of glycosylation and dimerization, can profoundly affect virulence in animals or cells.

Mutations in E^rns^ that abrogate RNase activity in CSFV lead to virus attenuation [21,114]. CSFV becomes attenuated after abolishing the NLG site in E^rns^ by residue N^2^ substitution [39]. Additionally, mutation of C^171^ in E^rns^ prevents homodimerization and also leads to the attenuation of CSFV [33].

When 19 amino acids are inserted into the *C*-terminus of E1, the highly virulent CSFV strain becomes completely attenuated [115]. Additionally, when amino acids N^6^, N^19^, and N^100^ of E1 are substituted, thus abolishing the NLG sites, the highly virulent CSFV strain loses its infectivity and becomes attenuated [40]. Substitution of cysteine residues at positions 24 and 94 in CSFV E1 affects E1-E2 heterodimerization and alters virulence [35].

The highly virulent CSFV strain is attenuated when the E2 gene is replaced, suggesting that E2 plays a major role in virulence [116]. Mutations in its *C*-terminal region can also influence its virulence [117]. The conserved epitope ^140^TAVSPTTLR^148^ in domain A plays an important role in virulence [118], and the misposition of T^140^ (T^141^ in CSFV vaccine strain GPE-) in this conserved epitope reduces CSFV virulence by influencing both virus replication efficiency *in vitro* and viral pathogenicity in pigs [119]. Mutations in two residues in E2, S^74^L and P^279^H, result in attenuation of the virus [120]; however, the E2 L^21^H mutation only attenuates the virus if there are additional mutations in residues R^9^, R^209^, and I^210^ in E^rns^ [121]. These mutations likely affect the interaction between E2 and E^rns^ during membrane anchoring. Glycosylation of E2 also influences virulence in swine, with residue N^116^ being involved in attenuation of the virulent parental virus and residue N^185^ being critical for virus viability [41].

Considering the types of cells that are susceptible to CSFV, it is unlikely that the attenuation in pathogenicity that is discussed above involves a change in cell tropism (*i.e*., “altered” tropism). Rather, it is more likely the result of “reduced” tropism, meaning that CSFV tropism of cell types remains unchanged with its attenuation, whereas its affinity or intensity is reduced. A similar phenomenon exists for CSFV live attenuated vaccine virus, which presents within the animal body in similar cell types but at a reduced intensity and for a shorter period of time post-infection. However, it is anticipated that the live attenuated vaccine virus contains alterations in glycoproteins as well as in other proteins.

### 4.2. Antigenic Differences Influence the Efficacy of E2 Subunit Vaccines

Because the genotypes of vaccine viruses (mostly genotypes 1.1 and 1.2) are different from those of the currently prevalent field viruses (genotype 2.1) [127], it is critical to clarify how antigenic differences influence cross-protection between vaccines and field isolates. Recombinant E2 proteins are effective against challenge with genotypically homologous CSFV strains [9,128,129,130,131,132] but do not offer complete protection against heterologous strains [9,133,134]. CSFV genotype-specific pig antisera bind to heterologous E2 proteins less efficiently than to homologous E2 proteins [135,136]. Additionally, antibodies that are induced by recombinant E2 proteins neutralize genotypically homologous strains better than heterologous strains [28]. All previous studies have indicated that the antigenic variation of E2 among CSFVs is crucial to cross-neutralization, which may explain the incomplete E2 vaccine protection with respect to heterologous strains [133].

### 4.3. Selection Pressure-Driven Mutations Determine the Appropriateness of Vaccines

Vaccination may affect strain diversity and immune escape through recombination events between vaccine strains and wild strains and through point mutations. Additionally, vaccination may influence the population dynamics, evolutionary rate, and adaptive evolution of CSFV [137].

The positive selection pressure that acts on the E^rns^, E1, and E2 envelope protein genes of CSFV has been studied to identify specific codons that are subjected to diversification. The selection for diversity likely occurs via two mechanisms, one of which is cell independent and the other of which is cell dependent. Selections of random mutations (e.g., 2.1 × 10^−^^2^ nucleotide substitution/site/year [138]) occur when extracellular virions are confronted with immunity, such as antibody- or cell-mediated immunity. Conversely, the selection of mutants in intracellular or cell membrane-associated virions or viral proteins would likely occur at an enhanced rate and via a more complicated mechanism because intracellular signaling would be involved in driving viral mutations, requiring at least one cycle of replication to correct lesions (mutations) and perpetuate viral diversification. No evidence for positive selection has been observed to date in E1.

The positively selected site at residue 209 of E^rns^ corresponds to an amino acid substitution from Ser to Arg that has been found in an HS-binding CSFV variant [108]. Four positively selected sites in E2 at residues 49, 72, 75, and 200 have been identified [108,109]. The mutant at residue 72 is responsible for antigenic specificity [54], and residue 75 is located within an O-glycosylation motif that alters the predicted glycosylation pattern of the protein [108]. Additionally, positive selective pressure has defined six residues at 34, 36, 49, 72, 87, and 88 in domains B/C of E2 [110]. As variation in a single amino acid mutation could substantially affect the antigenicity of E2 [26,54,135], positive selective pressure may influence the cross-neutralization activities of vaccines (see above). Because important antigen-specific residues contribute to neutralization and because the positively selected sites were primarily identified as being located within the highly variable E2 B/C domains [26,54,108,109,110,135], these domains should represent main targets that are amenable to antigenic evolution under selection pressure imposed by vaccine immunity. These domains are also associated with strong reductions in neutralizing titers of the heterologous virus.

A previous study on the BVDV E2 gene identified five positively selected sites, at residues 194, 196, 213, 252, and 254 [111], all located at the *C*-terminus of E2. These sites that are found in BVDV are opposite to those found in CSFV, in which positive selected sites are defined at the *N*-terminus, are surface-exposed, and are therefore prime targets for host antibody responses. These contradictory results suggest that selection to avoid antibody recognition has not been a major factor in BVDV.

## 5. Concluding Remarks

As reviewed above, the glycoproteins of pestiviruses clearly possess a limited number of domains, three to four (Figure 1), and it is interesting to note how these glycoproteins can adapt and increase in complexity to fulfill a great number of different functions (Table 1). Pestiviruses contain a maximum of three glycoproteins, including several major proteins, such as E2, and others that serve as accessory proteins, such as E^rns^ and/or E1. Disulfide bonds are important structural components of pestivirus that are involved in the following processes: (1) maintaining the stable intramolecular conformation of E2; (2) dimerization, such as E^rns^ and E2 homodimerization and E1-E2 heterodimerization, which are important during viral entry and infectivity; and (3) increasing the number of epitopes, particularly conformational epitopes, on glycoproteins, such as those on E2. Glycosylation is another important aspect of the pestivirus life cycle, and N-glycosylation is important for the following processes: (1) transport and secretion; (2) the synthesis and processing of glycoproteins; (3) blocking of the induction of IFN, thereby evading innate immunity; and (4) induction of protective neutralizing antibodies. Variations in amino acids and antigenic structures also serve as strategies for increasing pestivirus complexity, either by random or selection pressure-driven point mutations or by recombination. The substitution of amino acids at key positions in pestivirus glycoproteins can profoundly affect (1) antigenic structures that interact with antibodies; (2) abrogation of disulfide bonds, the importance of which is described above; (3) glycosylation; and (4) virion viability. Collectively, pestiviruses’ glycoproteins possess multiple functions and play critical roles in virus replication and pathogenicity.

## References

[1] Simmonds P., Becher P., Collett M., Gould E., Heinz F., Meyers G., Monath T., Pletnev A., Rice C., Stiasny K., King A.M.Q., Adams M.J., Carstens E.B. (2011). Virus Taxonomy: Ninth Report of the International Committee on Taxonomy of Viruses.

[2] Lindenbach B.D., Thiel H.J., Rice C.M., Knipe D.M., Howley P.M. (2007). Flaviviridae: The viruses and their replication. Fields Virology.

[3] Becher P., Avalos Ramirez R., Orlich M., Cedillo Rosales S., Konig M., Schweizer M., Stalder H., Schirrmeier H., Thiel H.J. (2003). Genetic and antigenic characterization of novel pestivirus genotypes: Implications for classification. Virology.

[4] Meyers G., Thiel H.J. (1996). Molecular characterization of pestiviruses. Adv. Virus Res..

[5] Thiel H.J., Stark R., Weiland E., Rümenapf T., Meyers G. (1991). Hog cholera virus: Molecular composition of virions from a pestivirus. J. Virol..

[6] Rümenapf T., Unger G., Strauss J.H., Thiel H.J. (1993). Processing of the envelope glycoproteins of pestiviruses. J. Virol..

[7] Bintintan I., Meyers G. (2010). A new type of signal peptidase cleavage site identified in an RNA virus polyprotein. J. Biol. Chem..

[8] Weiland E., Stark R., Haas B., Rümenapf T., Meyers G., Thiel H.J. (1990). Pestivirus glycoprotein which induces neutralizing antibodies forms part of a disulfide-linked heterodimer. J. Virol..

[9] König M., Lengsfeld T., Pauly T., Stark R., Thiel H.J. (1995). Classical swine fever virus: Independent induction of protective immunity by two structural glycoproteins. J. Virol..

[10] Weiland E., Ahl R., Stark R., Weiland F., Thiel H.J. (1992). A second envelope glycoprotein mediates neutralization of a pestivirus, hog cholera virus. J. Virol..

[11] Hulst M.M., Himes G., Newbigin E., Moormann R.J. (1994). Glycoprotein E2 of classical swine fever virus: Expression in insect cells and identification as a ribonuclease. Virology.

[12] Schneider R., Unger G., Stark R., Schneider-Scherzer E., Thiel H.J. (1993). Identification of a structural glycoprotein of an RNA virus as a ribonuclease. Science.

[13] Fetzer C., Tews B.A., Meyers G. (2005). The carboxy-terminal sequence of the pestivirus glycoprotein E^rns^ represents an unusual type of membrane anchor. J. Virol..

[14] Tews B.A., Meyers G. (2007). The pestivirus glycoprotein E^rns^ is anchored in plane in the membrane via an amphipathic helix. J. Biol. Chem..

[15] Aberle D., Muhle-Goll C., Bürck J., Wolf M., Reißer S., Luy B., Wenzel W., Ulrich A.S., Meyers G. (2014). Structure of the membrane anchor of pestivirus glycoprotein E^rns^, a long tilted amphipathic helix. PLoS Pathog..

[16] Burrack S., Aberle D., Bürck J., Ulrich A.S., Meyers G. (2012). A new type of intracellular retention signal identified in a pestivirus structural glycoprotein. FASEB J..

[17] Hulst M.M., van Gennip H.G., Moormann R.J. (2000). Passage of classical swine fever virus in cultured swine kidney cells selects virus variants that bind to heparan sulfate due to a single amino acid change in envelope protein E^rns^. J. Virol..

[18] Iqbal M., McCauley J.W. (2002). Identification of the glycosaminoglycan binding site on the glycoprotein E^rns^ of bovine viral diarrhoea virus by site directed mutagenesis. J. Gen. Virol..

[19] Krey T., Bontems F., Vonrhein C., Vaney M.C., Bricogne G., Rümenapf T., Rey F.A. (2012). Crystal structure of the pestivirus envelope glycoprotein E^rns^ and mechanistic analysis of its ribonuclease activity. Structure.

[20] Hulst M., Panoto F., Hoekman A., van Gennip H., Moormann R. (1998). Inactivation of the RNase activity of glycoprotein E^rns^ of classical swine fever virus results in a cytopathogenic virus. J. Virol..

[21] Meyers G., Saalmüller A., Büttner M. (1999). Mutations abrogating the RNase activity in glycoprotein E^rns^ of the pestivirus classical swine fever virus lead to virus attenuation. J. Virol..

[22] Wang J., Li Y., Modis Y. (2014). Structural models of the membrane anchors of envelope glycoproteins E1 and E2 from pestiviruses. Virology.

[23] Wensvoort G. (1989). Topographical and functional mapping of epitopes on hog cholera virus with monoclonal antibodies. J. Gen. Virol..

[24] Wensvoort G., Boonstra J., Bodzinga B.G. (1990). Immunoaffinity purification and characterization of the envelope protein E1 of hog cholera virus. J. Gen. Virol..

[25] Chang C.Y., Huang C.C., Lin Y.J., Deng M.C., Chen H.C., Tsai C.H., Chang W.M., Wang F.I. (2010). Antigenic domains analysis of classical swine fever virus E2 glycoprotein by mutagenesis and conformation-dependent monoclonal antibodies. Virus Res..

[26] Chang C.Y., Huang C.C., Deng M.C., Huang Y.L., Lin Y.J., Liu H.M., Lin Y.L., Wang F.I. (2012). Identification of conformational epitopes and antigen-specific residues at the D/A domains and the extramembrane *C*-terminal region of E2 glycoprotein of classical swine fever virus. Virus Res..

[27] Van Rijn P.A., Miedema G.K.W., Wensvoort G., van Gennip H.G.P., Moormann R.J.M. (1994). Antigenic structure of envelope glycoprotein E1 of hog cholera virus. J. Virol..

[28] Huang Y.L., Deng M.C., Wang F.I., Huang C.C., Chang C.Y. (2014). The challenges of classical swine fever control: Modified live and E2 subunit vaccines. Virus Res..

[29] El Omari K., Iourin O., Harlos K., Grimes J.M., Stuart D.I. (2013). Structure of a pestivirus envelope glycoprotein E2 clarifies its role in cell entry. Cell Rep..

[30] Li Y., Wang J., Kanai R., Modis Y. (2013). Crystal structure of glycoprotein E2 from bovine viral diarrhea virus. Proc. Natl. Acad. Sci..

[31] Iourin O., Harlos K., El Omari K., Lu W., Kadlec J., Iqbal M., Meier C., Palmer A., Jones I., Thomas C. (2013). Expression, purification and crystallization of the ectodomain of the envelope glycoproteinE2 from Bovine viral diarrhoea virus. Acta. Crystallogr. Sect. F Struct. Biol. Cryst. Commun..

[32] Langedijk J., van Veelen P., Schaaper W., de Ru A., Meloen R., Hulst M. (2002). A structural model of pestivirus E^rns^ based on disulfide bond connectivity and homology modeling reveals an extremely rare vicinal disulfide. J. Virol..

[33] Tews B.A., Schürmann E.M., Meyers G. (2009). Mutation of cysteine 171 of pestivirus E^rns^ RNase prevents homodimer formation and leads to attenuation of classical swine fever virus. J. Virol..

[34] Van Gennip H.G., Hesselink A.T., Moormann R.J., Hulst M.M. (2005). Dimerization of glycoprotein E^rns^ of classical swine fever virus is not essential for viral replication and infection. Arch. Virol..

[35] Fernández-Sainz I., Holinka L.G., Gladue D., O’Donnell V., Lu Z., Gavrilov B.K., Risatti G.R., Borca M.V. (2011). Substitution of specific cysteine residues in the E1 glycoprotein of classical swine fever virus strain Brescia affects formation of E1-E2 heterodimers and alters virulence in swine. J. Virol..

[36] Ronecker S., Zimmer G., Herrler G., Greiser-Wilke I., Grummer B. (2008). Formation of bovine viral diarrhea virus E1-E2 heterodimers is essential for virus entry and depends on charged residues in the transmembrane domains. J. Gen. Virol..

[37] Kornfeld R., Kornfeld S. (1985). Assembly of asparagine-linked oligosaccharides. Annu. Rev. Biochem..

[38] Branza-Nichita N., Lazar C., Dwek R.A., Zitzmann N. (2004). Role of N-glycan trimming in the folding and secretion of the pestivirus protein E^rns^. Biochem. Biophys. Res. Commun..

[39] Sainz I.F., Holinka L.G., Lu Z., Risatti G.R., Borca M.V. (2008). Removal of a N-linked glycosylation site of classical swine fever virus strain Brescia E^rns^ glycoprotein affects virulence in swine. Virology.

[40] Fernandez-Sainz I., Holinka L.G., Gavrilov B.K., Prarat M.V., Gladue D., Lu Z., Jia W., Risatti G.R., Borca M.V. (2009). Alteration of the N-linked glycosylation condition in E1 glycoprotein of classical swine fever virus strain Brescia alters virulence in swine. Virology.

[41] Risatti G., Holinka L., Sainz I.F., Carrillo C., Lu Z., Borca M. (2007). N-linked glycosylation status of classical swine fever virus strain Brescia E2 glycoprotein influences virulence in swine. J. Virol..

[42] Tyborowska J., Zdunek E., Szewczyk B. (2007). Effect of N-glycosylation inhibition on the synthesis and processing of classical swine fever virus glycoproteins. Acta. Biochim. Pol..

[43] Luo X., Pan R., Wan C., Liu X., Wu J., Pan Z. (2009). Glycosylation of classical swine fever virus E^rns^ is essential for binding double-stranded RNA and preventing interferon-beta induction. Virus Res..

[44] Gavrilov B.K., Rogers K., Fernandez-Sainz I.J., Holinka L.G., Borca M.V., Risatti G.R. (2011). Effects of glycosylation on antigenicity and immunogenicity of classical swine fever virus envelope proteins. Virology.

[45] Langedijk J.P.M., Middel W.G.J., Meloen R.H., Kramps J.A., de Smit J.A. (2001). Enzyme-linked immunosorbent assay using a virus type-specific peptide based on a subdomain of envelope protein E^rns^ for serologic diagnosis of pestivirus infections in swine. J. Clin. Microbiol..

[46] Lin M., Trottier E., Pasick J., Sabara M. (2004). Identification of antigenic regions of the E^rns^ protein for pig antibodies elicited during classical swine fever virus infection. J. Biochem..

[47] Lin M., McRae H., Dan H., Tangorra E., Laverdiere A., Pasick J. (2010). High-resolution epitope mapping for monoclonal antibodies to the structural protein E^rns^ of classical swine fever virus using peptide array and random peptide phage display approaches. J. Gen. Virol..

[48] Christmann A., Wentzel A., Meyer C., Meyers G., Kolmar H. (2001). Epitope mapping and affinity purification of monospecific antibodies by Escherichia coli cell surface display of gene-derived random peptide libraries. J. Immunol. Methods.

[49] Zhang F., Yu M., Weiland E., Morrissy C.J., Zhang N., Westbury H.A., Wang L.F. (2006). Characterization of epitopes for neutralizing monoclonal antibodies to classical swine fever virus E2 and E^rns^ using phage-displayed random peptide library. Arch. Virol..

[50] Meyer D., Aebischer A., Müller M., Grummer B., Greiser-Wilke I., Moennig V., Hofmann M.A. (2012). New insights into the antigenic structure of the glycoprotein E^rns^ of classical swine fever virus by epitope mapping. Virology.

[51] Van Rijn P.A., van Gennip H.G.P., de Meijer E.J., Moormann R.J.M. (1992). A preliminary map of epitopes on envelope glycoprotein El of HCV strain Brescia. Vet. Microbiol..

[52] Van Rijn P.A., van Gennip H.G.P., de Meijer E.J., Moormann R.J.M. (1993). Epitope mapping of envelope glycoprotein El of hog cholera virus strain Brescia. J. Gen. Virol..

[53] Chang C.Y., Huang C.C., Deng M.C., Huang Y.L., Lin Y.J., Liu H.M., Lin Y.L., Wang F.I. (2012). Antigenic mimicking with cysteine-based cyclized peptides reveals a previously unknown antigenic determinant on E2 glycoprotein of classical swine fever virus. Virus Res..

[54] Chang C.Y., Huang C.C., Lin Y.J., Deng M.C., Tsai C.H., Chang W.M., Wang F.I. (2010). Identification of antigen-specific residues on E2 glycoprotein of classical swine fever virus. Virus Res..

[55] Van Rijn P.A. (2007). A common neutralizing epitope on envelope glycoprotein E2 of different pestiviruses: Implications for improvement of vaccines and diagnostics for classical swine fever (CSF)?. Vet. Microbiol..

[56] Peng W.P., Hou Q., Xia Z.H., Chen D., Li N., Sun Y., Qiu H.J. (2008). Identification of a conserved linear B-cell epitope at the *N*-terminus of the E2 glycoprotein of classical swine fever virus by phage-displayed random peptide library. Virus Res..

[57] Tong C., Chen N., Liao X., Xie W., Li D., Li X., Fang W. (2015). The epitope recognized by monoclonal antibody 2B6 in the B/C domains of classical swine fever virus glycoprotein E2 affects binding to hyperimmune sera and virus replication. J. Microbiol. Biotechnol..

[58] Lin M., Lin F., Mallory M., Clavijo A. (2000). Deletions of structural glycoprotein E2 of classical swine fever virus strain Alfort/187 resolve a linear epitope of monoclonal antibody WH303 and the minimal *N*-terminal domain essential for binding immunoglobulin G antibodies of a pig hyperimmune serum. J. Virol..

[59] Kortekaas J., Vloet R.P.M., Weerdmeester K., Ketelaar J., van Eijk M., Loeffen W.L. (2010). Rational design of a classical swine fever C-strain vaccine virus that enables the differentiation between infected and vaccinated animals. J. Virol. Methods.

[60] Yu M., Wang L.F., Shiell B.J., Morrissy C.J., Westbury H.A. (1996). Fine mapping of a *C*-terminal linear epitope highly conserved among the major envelope glycoprotein E2 (gp51 to gp54) of different pestiviruses. Virology.

[61] Li G.X., Zhou Y.J., Yu H., Li L., Wang Y.X., Tong W., Hou J.W., Xu Y.Z., Zhu J.P., Xu A.T. (2012). A novel dendrimeric peptide induces high level neutralizing antibodies against classical swine fever virus in rabbits. Vet. Microbiol..

[62] Liu S., Tu C., Wang C., Yu X., Wu J., Guo S., Shao M., Gong Q., Zhu Q., Kong X. (2006). The protective immune response induced by B cell epitope of classical swine fever virus glycoprotein E2. J. Virol. Methods.

[63] Liu S., Yu X., Wang C., Wu J., Kong X., Tu C. (2006). Quadruple antigenic epitope peptide producing immune protection against classical swine fever virus. Vaccine.

[64] Monsó M., Tarradas J., de la Torre B.G., Sobrino F., Ganges L., Andreu D. (2011). Peptide vaccine candidates against classical swine fever virus: T cell and neutralizing antibody responses of dendrimers displaying E2 and NS2–3 epitopes. J. Pept. Sci..

[65] Reimann I., Depner K., Utke K., Leifer I., Lange E., Beer M. (2010). Characterization of a new chimeric marker vaccine candidate with a mutated antigenic E2-epitope. Vet. Microbiol..

[66] Tarradas J., Monsó M., Muñoz M., Rosell R., Fraile L., Frías M.T., Domingo M., Andreu D., Sobrino F., Ganges L. (2011). Partial protection against classical swine fever virus elicited by dendrimeric vaccine-candidate peptides in domestic pigs. Vaccine.

[67] Qi Y., Zhang B.Q., Shen Z., Chen Y.H. (2009). Antigens containing TAVSPTTLR tandem repeats could be used in assaying antibodies to classical swine fever virus. Acta Virol..

[68] Paton D.J., Lowings J.P., Barrett A.D. (1992). Epitope mapping of the gp53 envelope protein of bovine viral diarrhea virus. Virology.

[69] Deregt D., van Rijn P.A., Wiens T.Y., van den Hurk J. (1998). Monoclonal antibodies to the E2 protein of a new genotype (type 2) of bovine viral diarrhea virus define three antigenic domains involved in neutralization. Virus Res..

[70] Jelsma H., Loeffen W.L., van Beuningen A., van Rijn P.A. (2013). Preliminary mapping of non-conserved epitopes on envelope glycoprotein E2 of Bovine viral diarrhea virus type 1 and 2. Vet. Microbiol..

[71] Deregt D., Bolin S.R., van den Hurk J., Ridpath J.F., Gilbert S.A. (1998). Mapping of a type 1-specific and a type-common epitope on the E2 (gp53) protein of bovine viral diarrhea virus with neutralization escape mutants. Virus Res..

[72] Hulst M.M., Moormann R.J. (1997). Inhibition of pestivirus infection in cell culture by envelope proteins E^rns^ and E2 of classical swine fever virus: E^rns^ and E2 interact with different receptors. J. Gen. Virol..

[73] Wang Z., Nie Y., Wang P., Ding M., Deng H. (2004). Characterization of classical swine fever virus entry by using pseudotyped viruses: E1 and E2 are sufficient to mediate viral entry. Virology.

[74] Liang D.L., Sainz I.F., Ansari I.H., Gil L.H., Vassilev V., Donis R.O. (2003). The envelope glycoprotein E2 is a determinant of cell culture tropism in ruminant pestiviruses. J. Gen. Virol..

[75] Reimann I., Depner K., Trapp S., Beer M. (2004). An avirulent chimeric Pestivirus with altered cell tropism protects pigs against lethal infection with classical swine fever virus. Virology.

[76] Asfor A.S., Wakeley P.R., Drew T.W., Paton D.J. (2014). Recombinant pestivirus E2 glycoproteins prevent viral attachment to permissive and non permissive cells with different efficiency. Virus Res..

[77] Hulst M.M., van Gennip H.G., Vlot A.C., Schooten E., de Smit A.J., Moormann R.J. (2001). Interaction of classical swine fever virus with membrane-associated heparan sulfate: Role for virus replication *in vivo* and virulence. J. Virol..

[78] Iqbal M., Flick-Smith H., McCauley J.W. (2000). Interactions of bovine viral diarrhoea virus glycoprotein E^rns^ with cell surface glycosaminoglycans. J. Gen. Virol..

[79] Chen J., He W.R., Shen L., Dong H., Yu J., Wang X., Yu S., Li Y., Li S., Luo Y. (2015). The laminin receptor is an attachment cellular receptor for classical swine fever virus. J. Virol..

[80] Maurer K., Krey T., Moennig V., Thiel H.J., Rümenapf T. (2004). CD46 is a cellular receptor for bovine viral diarrhea virus. J. Virol..

[81] Dräger C., Beer M., Blome S. (2015). Porcine complement regulatory protein CD46 and heparan sulfates are the major factors for classical swine fever virus attachment *in vitro*. Arch. Virol..

[82] Krey T., Himmelreich A., Heimann M., Menge C., Thiel H.J., Maurer K., Rümenapf T. (2006). Function of bovine CD46 as a cellular receptor for bovine viral diarrhea virus is determined by complement control protein 1. J. Virol..

[83] Zezafoun H., Decreux A., Desmecht D. (2011). Genetic and splice variations of Bostaurus CD46 shift cell permissivity to BVDV, the bovine pestivirus. Vet. Microbiol..

[84] Li X., Wang L., Zhao D., Zhang G., Luo J., Deng R., Yang Y. (2011). Identification of host cell binding peptide from an overlapping peptide library for inhibition of classical swine fever virus infection. Virus Genes.

[85] Schelp C., Greiser-Wilke I., Moennig V. (2000). An actin-binding protein is involved in pestivirus entry into bovine cells. Virus Res..

[86] He F., Ling L., Liao Y., Li S., Han W., Zhao B., Sun Y., Qiu H.J. (2014). Beta-actin interacts with the E2 protein and is involved in the early replication of classical swine fever virus. Virus Res..

[87] Yang Z., Shi Z., Guo H., Qu H., Zhang Y., Tu C. (2015). Annexin 2 is a host protein binding to classical swine fever virus E2 glycoprotein and promoting viral growth in PK-15 cells. Virus Res..

[88] Gladue D.P., Baker-Bransetter R., Holinka L.G., Fernandez-Sainz I.J., O’Donnell V., Fletcher P., Lu Z., Borca M.V. (2014). Interaction of CSFV E2 protein with swine host factors as detected by yeast two-hybrid system. PLoS ONE.

[89] Garry R.F., Dash S. (2003). Proteomics computational analyses suggest that hepatitis C virus E1 and pestivirus E2 envelope glycoproteins are truncated class II fusion proteins. Virology.

[90] Fernández-Sainz I., Largo E., Gladue D., Fletcher P., O’Donnell V., Holinka L., Carey L., Lu X., Nieva J., Borca M. (2014). Effect of specific amino acid substitutions in the putative fusion peptide of structural glycoprotein E2 on classical swine fever virus replication. Virology.

[91] Grummer B., Grotha S., Greiser-Wilke I. (2004). Bovine viral diarrhoea virus is internalized by clathrin-dependent receptor mediated endocytosis. J. Vet. Med. B Infect. Dis. Vet. Public Health.

[92] Lecot S., Belouzard S., Dubuisson J., Rouillé Y. (2005). Bovine viral diarrhea virus entry is dependent on clathrin-mediated endocytosis. J. Virol..

[93] Krey T., Thiel H.J., Rümenapf T. (2005). Acid-resistant bovine pestivirus requires activation for pH-triggered fusion during entry. J. Virol..

[94] Kampmann T., Mueller D.S., Mark A.E., Young P.R., Kobe B. (2006). The Role of histidine residues in low-pH-mediated viral membrane fusion. Structure.

[95] Zürcher C., Sauter K.S., Mathys V., Wyss F., Schweizer M. (2014). Prolonged activity of the pestiviral RNase E^rns^ as an interferon antagonist after uptake by clathrin-mediated endocytosis. J. Virol..

[96] Fu Q., Shi H., Ren Y., Guo F., Ni W., Qiao J., Wang P., Zhang H., Chen C. (2014). Bovine viral diarrhea virus infection induces autophagy in MDBK cells. J. Microbiol..

[97] Pei J., Zhao M., Ye Z., Gou H., Wang J., Yi L., Dong X., Liu W., Luo Y., Liao M. (2014). Autophagy enhances the replication of classical swine fever virus *in vitro*. Autophagy.

[98] Fu Q., Shi H., Shi M., Meng L., Bao H., Zhang G., Ren Y., Zhang H., Guo F., Qiao J. (2014). Roles of bovine viral diarrhea virus envelope glycoproteins in inducing autophagy in MDBK cells. Microb. Pathog..

[99] Branza-Nichita N., Durantel D., Carrouée-Durantel S., Dwek R.A., Zitzmann N. (2001). Antiviral effect of N-butyldeoxynojirimycin against bovine viral diarrhea virus correlates with misfolding of E2 envelope proteins and impairment of their association into E1-E2 heterodimers. J. Virol..

[100] Armengol E., Wiesmüller K.H., Wienhold D., Büttner M., Pfaff E., Jung G., Saalmüller A. (2002). Identification of T-cell epitopes in the structural and non-structural proteins of classical swine fever virus. J. Gen. Virol..

[101] Ceppi M., de Bruin M.G., Seuberlich T., Balmelli C., Pascolo S., Ruggli N., Wienhold D., Tratschin J.D., McCullough K.C., Summerfield A. (2005). Identification of classical swine fever virus protein E2 as a target for cytotoxic T cells by using mRNA-transfected antigen-presenting cells. J. Gen. Virol..

[102] Franzoni G., Kurkure N.V., Essler S.E., Pedrera M., Everett H.E., Bodman-Smith K.B., Crooke H.R., Graham S.P. (2013). Proteome-wide screening reveals immunodominance in the CD8 T cell response against classical swine fever virus with antigen-specificity dependent on MHC class I haplotype expression. PLoS ONE.

[103] Bruschke C.J., Hulst M.M., Moormann R.J., van Rijn P.A., van Oirschot J.T. (1997). Glycoprotein E^rns^ of pestiviruses induces apoptosis in lymphocytes of several species. J. Virol..

[104] Magkouras I., Mätzener P., Rümenapf T., Peterhans E., Schweizer M. (2008). RNase-dependent inhibition of extracellular, but not intracellular, dsRNA-induced interferon synthesis by E^rns^ of pestiviruses. J. Gen. Virol..

[105] Mätzener P., Magkouras I., Rümenapf T., Peterhans E., Schweizer M. (2009). The viral RNase E^rns^ prevents IFN type-I triggering by pestiviral single- and double-stranded RNAs. Virus Res..

[106] Meyers G., Ege A., Fetzer C., von Freyburg M., Elbers K., Carr V., Prentice H., Charleston B., Schürmann E.M. (2007). Bovine viral diarrhea virus: Prevention of persistent fetal infection by a combination of two mutations affecting E^rns^ RNase and N^pro^ protease. J. Virol..

[107] Python S., Gerber M., Suter R., Ruggli N., Summerfield A. (2013). Efficient sensing of infected cells in absence of virus particles by plasmacytoid dendritic cells is blocked by the viral ribonuclease E^rns^. PLoS Pathog..

[108] Tang F., Pan Z., Zhang C. (2008). The selection pressure analysis of classical swine fever virus envelope protein genes Erns and E2. Virus Res..

[109] Shen H., Pei J., Bai J., Zhao M., Ju C., Yi L., Kang Y., Zhang X., Chen L., Li Y. (2011). Genetic diversity and positive selection analysis of classical swine fever virus isolates in south China. Virus Genes.

[110] Pérez L.J., Díaz de Arce H., Perera C.L., Rosell R., Frías M.T., Percedo M.I., Tarradas J., Dominguez P., Núñez J.I., Ganges L. (2012). Positive selection pressure on the B/C domains of the E2-gene of classical swine fever virus in endemic areas under C-strain vaccination. Infect. Genet. Evol..

[111] Tang F., Zhang C. (2007). Evidence for positive selection on the E2 gene of bovine viral diarrhoea virus type 1. Virus Genes.

[112] Leifer I., Ruggli N., Blome S. (2013). Approaches to define the viral genetic basis of classical swine fever virus virulence. Virology.

[113] Ji W., Guo Z., Ding N., He C. (2015). Studying classical swine fever virus: Making the best of a bad virus. Virus Res..

[114] Meyer C., von Freyburg M., Elbers K., Meyers G. (2002). Recovery of virulent and RNase-negative attenuated type 2 bovine viral diarrhea viruses from infectious cDNA clones. J. Virol..

[115] Risatti G., Holinka L., Lu Z., Kutish G., Tulman E., French R., Sur J., Rock D., Borca M. (2005). Mutation of E1 glycoprotein of classical swine fever virus affects viral virulence in swine. Virology.

[116] Risatti G., Borca M., Kutish G., Lu Z., Holinka L., French R., Tulman E., Rock D. (2005). The E2 glycoprotein of classical swine fever virus is a virulence determinant in swine. J. Virol..

[117] Risatti G., Holinka L., Fernandez Sainz I., Carrillo C., Kutish G., Lu Z., Zhu J., Rock D., Borca M. (2007). Mutations in the carboxyl terminal region of E2 glycoprotein of classical swine fever virus are responsible for viral attenuation in swine. Virology.

[118] Risatti G., Holinka L., Carrillo C., Kutish G., Lu Z., Tulman E., Sainz I.F., Borca M. (2006). Identification of a novel virulence determinant within the E2 structural glycoprotein of classical swine fever virus. Virology.

[119] Tamura T., Sakoda Y., Yoshino F., Nomura T., Yamamoto N., Sato Y., Okamatsu M., Ruggli N., Kida H. (2012). Selection of classical swine fever virus with enhanced pathogenicity reveals synergistic virulence determinants in E2 and NS4B. J. Virol..

[120] Fahnøe U., Pedersen A.G., Risager P.C., Nielsen J., Belsham G.J., Höper D., Beer M., Rasmussen T.B. (2014). Rescue of the highly virulent classical swine fever virus strain “Koslov” from cloned cDNA and first insights into genome variations relevant for virulence. Virology.

[121] Van Gennip H.G., Vlot A.C., Hulst M.M., de Smit A.J., Moormann R.J. (2004). Determinants of virulence of classical swine fever virus strain Brescia. J. Virol..

[122] Branza-Nichita N., Lazar C., Durantel D., Dwek R.A., Zitzmann N. (2002). Role of disulfide bond formation in the folding and assembly of the envelope glycoproteins of a pestivirus. Biochem. Biophys. Res. Commun..

[123] Aebischer A., Müller M., Hofmann M.A. (2013). Two newly developed E^rns^-based ELISAs allow the differentiation of Classical Swine Fever virus-infected from marker-vaccinated animals and the discrimination of pestivirus antibodies. Vet. Microbiol..

[124] Cheng C.Y., Wu C.W., Lin G.J., Lee W.C., Chien M.S., Huang C. (2014). Enhancing expression of the classical swine fever virus glycoprotein E2 in yeast and its application to a blocking ELISA. J. Biotechnol..

[125] Schroeder S., von Rosen T., Blome S., Loeffen W., Haegeman A., Koenen F., Uttenthal Å. (2012). Evaluation of classical swine fever virus antibody detection assays with an emphasis on the differentiation of infected from vaccinated animals. Rev. Sci. Tech. Off. Int. Epiz..

[126] Windisch J.M., Schneider R., Stark R., Weiland E., Meyers G., Thiel H.J. (1996). RNase of classical swine fever virus: Biochemical characterization and inhibition by virus-neutralizing monoclonal antibodies. J. Virol..

[127] Chen N., Hu H., Zhang Z., Shuai J., Jiang L., Fang W. (2008). Genetic diversity of the envelope glycoprotein E2 of classical swine fever virus: Recent isolates branched away from historical and vaccine strains. Vet. Microbiol..

[128] Bouma A., de Smit A.J., de Kluijver E.P., Terpstra C., Moormann R.J.M. (1999). Efficacy and stability of a subunit vaccine based on glycoprotein E2 of classical swine fever virus. Vet. Microbiol..

[129] Bouma A., de Smit A.J., de Jong M.C.M., de Kluijver E.P., Moormann R.J.M. (2000). Determination of the onset of the herd-immunity induced by the E2 sub-unit vaccine against classical swine fever virus. Vaccine.

[130] Hulst M.M., Westra D.F., Wensvoort G., Moormann R.J. (1993). Glycoprotein E1 of hog cholera virus expressed in insect cells protects swine from hog cholera. J. Virol..

[131] Rümenapf T., Stark R., Meyers G., Thiel H.J. (1991). Structural proteins of hog cholera virus expressed by vaccinia virus: Further characterization and induction of protective immunity. J. Virol..

[132] Van Zijl M., Wensvoort G., de Kluyver E., Hulst M., van der Gulden H., Gielkens A., Berns A., Moormann R. (1991). Live attenuated pseudorabies virus expressing envelope glycoprotein E1 of hog cholera virus protects swine against both pseudorabies and hog cholera. J. Virol..

[133] Uttenthal Å., le Potier M.F., Romero L., de Mia G.M., Floegel-Niesmann G. (2001). Classical swine fever (CSF) marker vaccine-Trial I. Challenge studies in weaner pigs. Vet. Microbiol..

[134] Van Oirschot J.T. (2003). Vaccinology of classical swine fever: From lab to field. Vet. Microbiol..

[135] Chen N., Tong C., Li D., Wan J., Yuan X., Li X., Peng J., Fang W. (2010). Antigenic analysis of classical swine fever virus E2 glycoprotein using pig antibodies identifies residues contributing to antigenic variation of the vaccine C-strain and group 2 strains circulating in China. Virol. J..

[136] Luo L., Nishi K., Macleod E., Sabara M.I., Lin M., Handel K., Pasick J. (2013). Baculovirus expression and antigenic characterization of classical swine fever virus E2 proteins. Transbound. Emerg. Dis..

[137] Ji W., Niu D.D., Si H.L., Ding N.Z., He C.Q. (2014). Vaccination influences the evolution of classical swine fever virus. Infect. Genet. Evol..

[138] De Arce H.D., Ganges L., Barrera M., Naranjo D., Sobrino F., Frías M.T., Núñez J.I. (2005). Origin and evolution of viruses causing classical swine fever in Cuba. Virus Res..

